# Evidence That the Periaqueductal Gray Matter Mediates the Facilitation of Panic-Like Reactions in Neonatally-Isolated Adult Rats

**DOI:** 10.1371/journal.pone.0090726

**Published:** 2014-03-03

**Authors:** Jeyce Willig Quintino-dos-Santos, Cláudia Janaína Torres Müller, Cristie Setúbal Bernabé, Caroline Azevedo Rosa, Sérgio Tufik, Luiz Carlos Schenberg

**Affiliations:** 1 Department of Physiological Sciences, Federal University of Espírito Santo, Vitória, ES, Brazil; 2 Department of Sports, Federal University of Espírito Santo, Vitória, ES, Brazil; 3 Department of Psychobiology, Federal University of São Paulo, São Paulo, SP, Brazil; INSERM/CNRS, France

## Abstract

Plenty of evidence suggests that childhood separation anxiety (CSA) predisposes the subject to adult-onset panic disorder (PD). As well, panic is frequently comorbid with both anxiety and depression. The brain mechanisms whereby CSA predisposes to PD are but completely unknown in spite of the increasing evidence that panic attacks are mediated at midbrain's dorsal periaqueductal gray matter (DPAG). Accordingly, here we examined whether the neonatal social isolation (NSI), a model of CSA, facilitates panic-like behaviors produced by electrical stimulations of DPAG of rats as adults. Eventual changes in anxiety and depression were also assessed in the elevated plus-maze (EPM) and forced-swimming test (FST) respectively. Male pups were subjected to 3-h daily isolations from post-natal day 2 (PN2) until weaning (PN21) allotting half of litters in individual boxes inside a sound-attenuated chamber (NSI, *n* = 26) whilst siblings (sham-isolated rats, SHAM, *n* = 27) and dam were moved to another box in a separate room. Non-handled controls (CTRL, *n* = 18) remained undisturbed with dams until weaning. As adults, rats were implanted with electrodes into the DPAG (PN60) and subjected to sessions of intracranial stimulation (PN65), EPM (PN66) and FST (PN67-PN68). Groups were compared by Fisher's exact test (stimulation sites), likelihood ratio chi-square tests (stimulus-response threshold curves) and Bonferroni's *post hoc* t-tests (EPM and FST), for P<0.05. Notably, DPAG-evoked panic-like responses of immobility, exophthalmus, trotting, galloping and jumping were markedly facilitated in NSI rats relative to both SHAM and CTRL groups. Conversely, anxiety and depression scores either did not change or were even reduced in neonatally-handled groups relative to CTRL, respectively. Data are the first behavioral evidence in animals that early-life separation stress produces the selective facilitation of panic-like behaviors in adulthood. Most importantly, results implicate the DPAG not only in panic attacks but also in separation-anxious children's predispositions to the late development of PD.

## Introduction

Dyspnoea, panic and urge to flee are the cardinal symptoms of clinical panic [1], [2]. Panic attacks are also precipitated by infusions of sodium lactate and inhalations of 5–7% carbon dioxide (CO_2_) in predisposed patients but not in healthy subjects [3], [4], [5]. These and other data led Donald Klein [1] to postulate that clinical panic is the misfiring of an evolved suffocation alarm system.

On the other hand, plenty evidence suggests that the periaqueductal gray matter (PAG) of the midbrain plays a crucial role in panic attacks [6], [7], [8], [9]. Indeed, while electrical stimulations of the dorsal half of PAG (DPAG) of humans produce panic, dyspnoea, chest pain and sensations of smothering or “hunger for air” [10], [11], the DPAG is markedly activated in both volunteers experiencing definite symptoms of smothering [12] and patients panicking upon the infusion of sodium lactate [13]. The DPAG is likewise activated during the exposure of volunteers to a virtual predator which was otherwise able to inflict real shocks to the subject's finger [14]. Curiously, as well, Amano and collaborators [15] had long reported that a patient stimulated in the PAG uttered “somebody is now chasing me, I'm trying to escape from him”. These observations are compatible with DPAG mediation of both the respiratory and non-respiratory types of panic attacks [16].

In rats, electrical and chemical stimulations of DPAG produces freezing and flight behaviors along with marked cardiorespiratory responses which are reminiscent of a panic attack [17], [18], [19], [20], [21]. Moreover, previous studies showed that DPAG-evoked defensive behaviors are either attenuated or virtually suppressed by chronic administrations of panicolytics in doses and regimens alike to those of the therapy of panic disorder (PD) [8]. Most notably, however, the recent study of Schimitel and colleagues [22] presented compelling evidence that the DPAG harbors a hypoxia-sensitive alarm system that may be implicated in both spontaneous and asphyxia-induced panic attacks. Consequently, the latter authors proposed that respiratory (suffocation-like) and non-respiratory (predation-like) panics are processed in the lateral (LPAG) and dorsolateral (DLPAG) regions of PAG respectively. The latter study was corroborated by c-fos immunohistochemistry data showing that the DPAG and the nucleus of solitary tract were both activated in rats escaping from 8% hypoxia [23]. The DPAG mediation of panic attacks was also supported by the recent report of CO_2_ provocation of panic in Urbach-Wiethe fear-unresponsive patients with bilateral extensive calcifications of the amygdala [24]. Accordingly, Feinstein and colleagues [24] concluded that panic is most likely mediated “at the brainstem” in spite of the established role of the amygdala in fear and anxiety of both humans and animals.

Clinical and epidemiological evidence suggests, on the other hand, that childhood separation anxiety (CSA) predisposes to the late development of PD [25], [26], [27]. In particular, twin-based clinical and epidemiological studies showed that CSA and PD share a common genetic diathesis [28], [29]. Moreover, separation-anxious offspring of parents with panic disorder (PD) presents ventilatory responses to hypercapnia similar to those observed in panic patients [30]. In the same vein, preclinical studies showed that respiratory responses to hypercapnia are facilitated in both mice and rats exposed to unstable familial environment (repeated cross-fostering) [31] and maternal separations [32], [33], [34], [35], [36] respectively. These and other results led Preter and Klein [37] to propose that CSA and PD are caused by a defective opioidergic buffering of both the separation and the suffocation alarm systems. Plenty evidence also suggests that early-life adverse events lead to neurobiological and behavioral manifestations of depression [38]. Indeed, panic is very often comorbid with depressive disorders [39], [40], [41]. Despite the above evidence, clinical and epidemiological recent studies reported that neither the CSA [27] nor the early-life adversity [28], [29] had any effect on the rate of depression and PD respectively. Accordingly, the influence of the early-life environment in the late development of panic and depression remains largely unsolved.

In any event, above studies suggest that CSA predisposing risk to adult-onset PD could be mediated by the DPAG. Thus, here we examined whether the neonatal social isolation (NSI), an experimental model of CSA, facilitates panic-like behaviors produced by electrical stimulations of DPAG of adult rats. As a corollary, NSI facilitation of DPAG-evoked defensive behaviors would be a compelling evidence of DPAG involvement in developmental aspects of PD. Eventual changes in anxiety and depression were also assessed in the elevated plus-maze (EPM) and forced-swimming test (FST) respectively.

## Materials

### Animals

Nulliparous pregnant Wistar rats were kept alone in polypropylene boxes (30 cm×20 cm×13 cm) until parturition. On postnatal day 2 (PN2), female pups were sacrificed with chloral hydrate (400 mg/kg, I.P.) and males were marked with a surgical pen (Texta Fineline 700, Japan) as isolated or non-isolated, according to the group to be allotted to. Except for the isolation periods, litters (maximum of 8 pups) remained with dams in larger polypropylene nest boxes (49 cm×34 cm×16 cm) up to weaning (PN21). Isolated and non-isolated siblings were raised separately in groups of 2 to 4 subjects in polypropylene boxes with food and water *ad libitum* and wood shave bedding. At the end of experiments, male rats were anesthetized with chloral hydrate and sacrificed concomitantly to the brain perfusion with saline (see *Histology*).

### Ethic statement

Experiments complied with the guidelines of the National Institute of Health Guide for the Care and Use of Laboratory Animals (NIH Publications No. 80-23, 1996) and were all approved by the local committee on the ethical use of animals in scientific research (Comitê de Ética no Uso de Animais da Escola de Medicina da Santa Casa de Misericórdia, Vitória, ES, CEUA-EMESCAM Protocol 023/2007).

### NSI procedure

In two groups, the NSI was carried out throughout the lactation period (PN2-PN21) according to a split-litter (twin-like) genetically-balanced design. Each morning half of pups of each litter (neonatally-isolated rats, NSI, n = 26) were allotted to individual boxes (30 cm×20 cm×13 cm) for a 3-h period whilst siblings (sham-isolated rats, SHAM, n = 27) and dam were moved to a novel box (49 cm×34 cm×16 cm) in a separate room. Environmental stimuli were attenuated by placing the isolation boxes inside a large incubator (49 cm×66 cm×96 cm) kept at room temperature (20–22°C) and having a roof opening (10 cm×10 cm) for air renewal. At the end of the isolation period, pups and dams were moved back to the nest box. In a third group, pups were kept undisturbed with dams up to weaning (control rats, CTRL, n = 18). Pups were handled by a single experimenter (JWQS) which was responsible for the cleaning of nest boxes at 5-day intervals. As adults, rats were implanted with electrodes aimed at the DPAG (PN60) and subjected to consecutive daily sessions of intracranial stimulation (PN65), EPM (PN66) and FST (training-session: PN67; test-session: PN68) (Table 1).

**Table 1:** Protocol of neonatal social isolation (NSI).

PN1	PN2-PN21	PN60	PN65	PN66	PN67	PN68	PN68
Sacrifice of female pups	3-h daily NSI	Surgery	ICS	EPM	FST-1	FST-2	Sacrifice

PN – postnatal day, ICS – intracranial stimulation, EPM – elevated plus-maze, FST-1 – forced swimming pretest session, FST-2 – forced swimming test session.

### Electrodes and surgery

Electrodes were made of a stainless steel wire (0.25 mm o.d.) (California Fine Wire Company, Grover City, USA) insulated throughout except at the cross section of the tip. A non-insulated stainless steel wire served as the indifferent electrode. Electrode implantation was carried out as previously described [20]. Thereafter, rats were allotted to glass-walled individual boxes (25 cm×15 cm×30 cm) with wood shave bedding and food and water *ad libitum*.

### DPAG stimulation

Five days after surgery, rats were stimulated in a Plexiglas cylindrical open-field (60 cm wall height and diameter) placed in a sound-attenuated temperature-controlled room (23–25°C). Stimulation was performed through a constant current sine-wave stimulator (FDV, Ribeirão Preto, Brazil) connected to a mercury swivel that allowed the free movement of the rat. Following a habituation period of 15 min, rats were stimulated with 30 s trains of stepwise increasing intensities (5 µA steps, 60 Hz, a.c.), applied 5 min apart.

### DPAG-evoked behavior recording

The DPAG-evoked ‘threshold responses’, i.e., the responses elicited with minimally effective currents, were recorded in a binary way, as emitted or not, irrespective of the response frequency or duration during a single stimulation trial. The rat defensive behaviors were recorded according to a statistically validated ethogram [20], as follows:

Exophthalmus – The eyes take on a spherical shape due to the eyeball protrusion and fully opening of the eyelid.
Immobility – Overall behavioral arrest accompanied by the increase in muscle tonus as suggested by the extension of neck and/or limbs and elevation of head, trunk and/or tail. Except for the visible tachypnoea, the rat looks like a ‘statue’ for periods as short as 3 s or lasting the whole stimulation trial (30 s). Tense immobility was invariably accompanied by exophthalmus, but not the inverse.
Trotting – Fast locomotion with out-of-phase stance and swing movements of contralateral limbs and the elevation of trunk and tail (not crawling).
Galloping – Running alternating stance and swing movements of anterior and posterior limb pairs.
Jumping – Upward leaps directed to the border of the open-field.
Defecation and micturition – Ejection of feces and urine (neither the search of a suitable place, nor the concealment of feces were reported in rats). The binary recording of threshold responses avoided the influence of colon and bladder emptying to repeated stimulations of DPAG.

### EPM procedure

The EPM was set 77 cm above the floor in a sound-attenuated temperature-controlled (23–25°C) low-lit (44 lux) room. The apparatus was a plus-shaped formica-covered wooden maze made up of 2 enclosed-arms (50×10 cm) surrounded by a 40-cm wall and 2 open-arms (50×10 cm) having a 1-cm aluminum ledge to minimize falls. Enclosed and open arms communicated through a central platform (10×10 cm). In the EPM procedure, the rat was placed in the central platform facing an enclosed arm and allowed to explore the maze for 5 min. Following each EPM session, the apparatus was cleaned with 10% ethyl alcohol solution. Sessions were filmed with a digital camera (SONY, CyberShot, Manaus, Brazil) and analyzed off-line by a single experimenter (JWQS). Anxiety-like behaviors were assessed through the percentage of entries (%OAE = 100× open arm entries/total arm entries) and time spent (%OAT = 100× open arm time/total arm time) in open arms. Exploratory activity was assessed through the number of enclosed arm entries (EAE). An ‘entry’ was defined as the invasion of the arm with four paws. The time spent in the central platform (CPT) was calculated as the session total duration (5 min) minus the time spent in arm exploration.

### FST procedure

The FST was carried out in a transparent plexiglas cylinder of 28.5 cm diameter and 62 cm height. In training session (FST-1), the cylinder was filled with water up to the height of 54 cm and the rat was forced to swim for 15 min. The day after (FST-2 test session), the rat was subjected to 5-min forced swimming session. FST test sessions were filmed with a digital camera and analyzed off-line. Floating duration was measured as the sum of periods in which the rat remained virtually immobile, except for the small movements to keep the head above the surface.

### Histology

After the terminus of experiments, rats were deeply anesthetized and intracardially perfused with the aid of a peristaltic pump (Masterflex C/L, model 77120-70, Barrington, USA) with 200 ml of 0.9% NaCl followed by 200 ml of 10% formaldehyde solution. Heads were further kept in 10% formaldehyde for a minimum of 4 days for the appropriate molding of the electrode tract. Thereafter, brains were removed, blocked and sectioned (60 µm) in a Cryostat (Leica CM 1850, Wetzlar, Germany). Sections were laid down onto glass slides, dried overnight (38°C), stained with neutral red (Sigma, St. Louis, USA) and mounted with DPX (Aldrich Chemical Company, Milwaukee, USA). Histological analysis was carried out through low magnification light microscopy (Leica DM 2500 microscope coupled to a DFC 300 FX camera, Wetzlar, Germany). Stimulation sites were plotted onto coronal diagrams of rat brain atlas [42].

### Statistics

#### Electrode localization

Group differences in electrode localization were assessed through Fisher's 2-tail exact test (P<0.05).

#### EPM and FST performances

Rat behaviors were compared by one-way ANOVA followed by *post hoc* t-tests for Bonferroni's 5% descriptive level.

#### DPAG-evoked responses

PAG-evoked responses were examined through the threshold logistic analysis. This method was devised as a convenient way to model the probabilities of intracranially-evoked unconditioned behaviors [20], [43]. Technically, the method is an extension of regression analysis of binary variables employed in the determination of median effective doses (ED_50_). As such, the method yields both the stimulus-response threshold curve (response probability distribution) and the population unbiased estimate of the median effective intensity (I_50_±SE).

Intensity-response curves were obtained by maximum likelihood fitting of response accumulated frequencies in function of the logarithm of current intensity, according to the logistic function, P(y_ij_|x_ij_) = [1+exp−(α_j_+β_j_x_ij_)]^−1^, where P is the expected probability of the response y_ij_ at a given stimulus x_ij_, α_j_ is the intercept and β_j_ the ‘slope’ (curvature parameter) of the *j*th curve (e.g., curves of NSI, SHAM or CTRL groups).

The population estimates of Log(I_50_) and I_50_ were computed as Log(I_50_) = −α_j_/β_j_ and I_50_ = 10^−αj/βj^ respectively. The standard error of Log(I_50_) was estimated as SE(LogI_50_) = ((Var(α)−2(α/β)Cov(α,β)+(α/β)^2^Var(β))/β^2^)^½^ [44]. Accordingly, the standard error of I_50_ was calculated as SE(I_50_) = I_50_(SE(LogI_50_)), where the parameter variances (Var) and covariances (Cov) were provided by the estimated covariance matrix of SAS logistic procedure.

Regression significant effects were assessed through Wald's chi-square, χ^2^_w_ = (β_j_/SE_β_)^2^ where ‘SE_β_’ is standard error of the curvature parameter (β_j_). Intensity-response (threshold) curves were parameterized through indicator variables (0, 1) and compared by likelihood-ratio χ^2^ tests [44]. Chi-square values were further partitioned to assess the net contribution of differences in either the location or slope of threshold curves. Curve pairwise comparisons (1 d.f.) were considered significant for Bonferroni's 5% descriptive level.

Statistical analyses were all performed with the SAS® statistical software (Statistical Analysis System, Cary, USA).

## Results

### Stimulated sites

**Figure 1:**
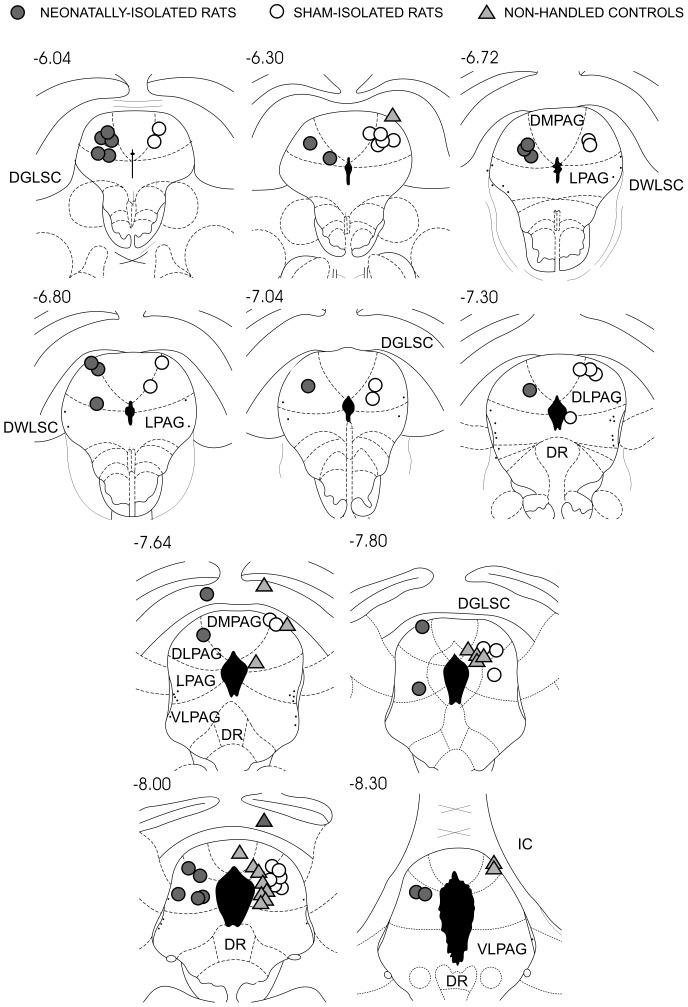
Localization of stimulating electrodes. Groups symbols were plotted in the same side for the sake of clarity. Abbreviations: DGLSC and DWLSC – deep gray and white layers of superior colliculus, DMPAG, DLPAG, LPAG and VLPAG – dorsomedial, dorsolateral, lateral and ventrolateral columns of the periaqueductal gray matter, DR – dorsal raphe, IC – inferior colliculus. Numbers refer to anteroposterior coordinates in relation to bregma of coronal plates of Paxinos and Watson's (1998) rat brain atlas [42].

Electrode localization did not differ significantly among groups (Fig. 1, Table 2). Thus, apart from 1 electrode in the dorsomedial column of PAG (DMPAG) and 3 electrodes in the deeper layers of superior colliculus (DLSC), electrodes were all localized into the DLPAG and LPAG. Moreover, LPAG electrodes were mostly localized at caudal levels of this column (−7.8 mm to −8.3 mm posterior to bregma).

**Table 2:** Brain areas stimulated in controls (CTRL), sham-isolated rats (SHAM) and neonatally-isolated rats (NSI).

	DMPAG		DLPAG		LPAG		DLSC		Total	
	n	*%*	n	%	n	*%*	n	*%*	n	*%*
CTRL	1	*5.5*	10	*55.5*	5	*27.7*	2	*11.1*	18	*25.4*
SHAM	–	–	20	*74.1*	7	*25.9*	–	–	27	*38.0*
NSI	–	–	17	*65.4*	8	*30.8*	1	*3.8*	26	*36.6*
Total	1	*1.4*	47	*66.2*	20	*28.2*	3	*4.2*	71	*100*

DMPAG, DLPAG and LPAG – dorsomedial, dorsolateral and lateral columns of periaqueductal gray matter, DLSC – deep layers of superior colliculus.

### Effects of NSI on the thresholds of DPAG-evoked responses

**Figure 2:**
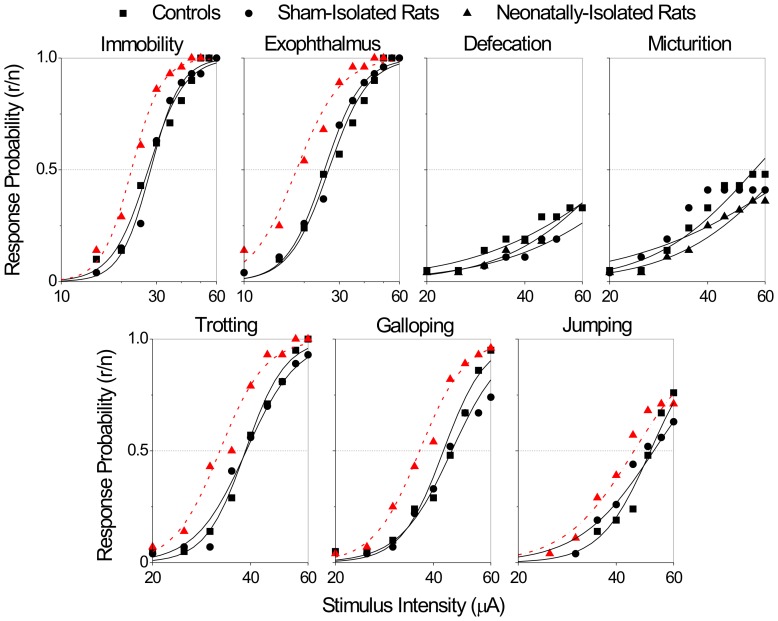
Intensity-response threshold curves of panic-like behaviors evoked by electrical stimulations of the dorsal periaqueductal gray matter. Curves are the best-fitted logistic functions of threshold response accumulated proportions in function of the logarithm of the stimulus intensity (µA). The abscissa is in logarithmic scale. Dashed lines (–) indicate significant differences between groups (P<0.05, likelihood-ratio χ^2^ test for curve location). Abbreviations: r – responders, n –number of stimulated rats.

**Figure 3:**
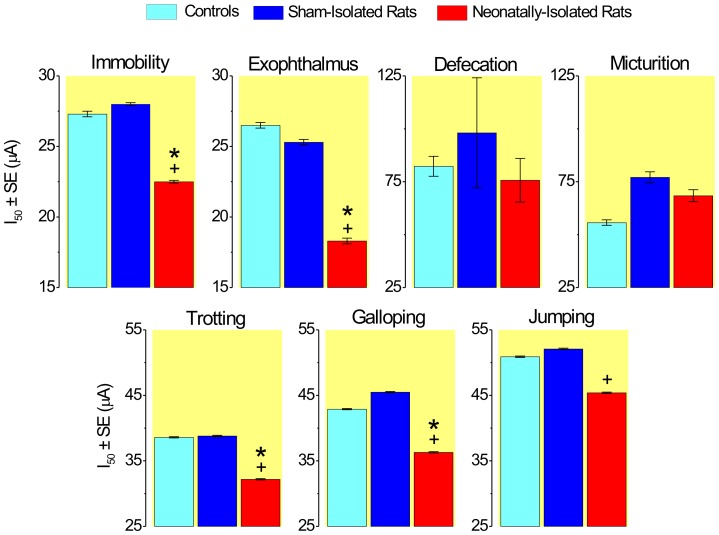
Median threshold intensities (I_50_±SE) of neonatally-isolated rats, sham-isolated rats and non-handled controls. Symbols represent values significantly different from controls (*) and sham-isolated rats (^+^) for Bonferroni's 5% criterion (likelihood ratio χ^2^ tests for curve location).

The overall comparison of threshold curves showed significant differences for immobility (χ^2^ = 23.5, 4 d.f., P<0.0001), exophthalmus (χ^2^ = 35.4, 4 d.f., P<0.0001), trotting (χ^2^ = 27.7, 4 d.f., P<0.0001), galloping (χ^2^ = 38.5, 4 d.f., P<0.0001) and jumping (χ^2^ = 12.3, 4 d.f., P<0.01) (Figures 2–3). Further partitioning of the χ^2^ showed that these differences were exclusively due to changes in the location (but not slope) of threshold curves. In contrast, micturition and defecation responses did not differ among groups.

Pairwise *post hoc* comparisons showed that the thresholds of NSI were significantly lower than those of CTRL for immobility (ΔI_50_% = −18%, χ^2^ = 13.8, 1 d.f., P<0.0005), exophthalmus (ΔI_50_% = −31%, χ^2^ = 26.0, 1 d.f., P<0.0001), trotting (ΔI_50_% = −17%, χ^2^ = 16.4, 1 d.f., P<0.0001) and galloping (ΔI_50_% = −16%, χ^2^ = 16.0, 1 d.f., P<0.0001), but not jumping (Figures 2–3).

As well, thresholds of NSI rats were significantly reduced as compared to those of SHAM rats for immobility (ΔI_50_% = −20%, χ^2^ = 19.3, 1 d.f., P<0.0001), exophthalmus (ΔI_50_% = −28%, χ^2^ = 22.7, 1 d.f., P<0.0001), trotting (ΔI_50_% = −17%, χ^2^ = 20.6, 1 d.f., P<0.0001), galloping (ΔI_50_% = −20%, χ^2^ = 33.6, 1 d.f., P<0.0001) and jumping (ΔI_50_% = −13%, χ^2^ = 7.7, 1 d.f., P<0.005) (Figures 2–3). Thresholds of CTRL and SHAM rat groups were but virtually identical.

### NSI effects on the EPM and FST

**Figure 4:**
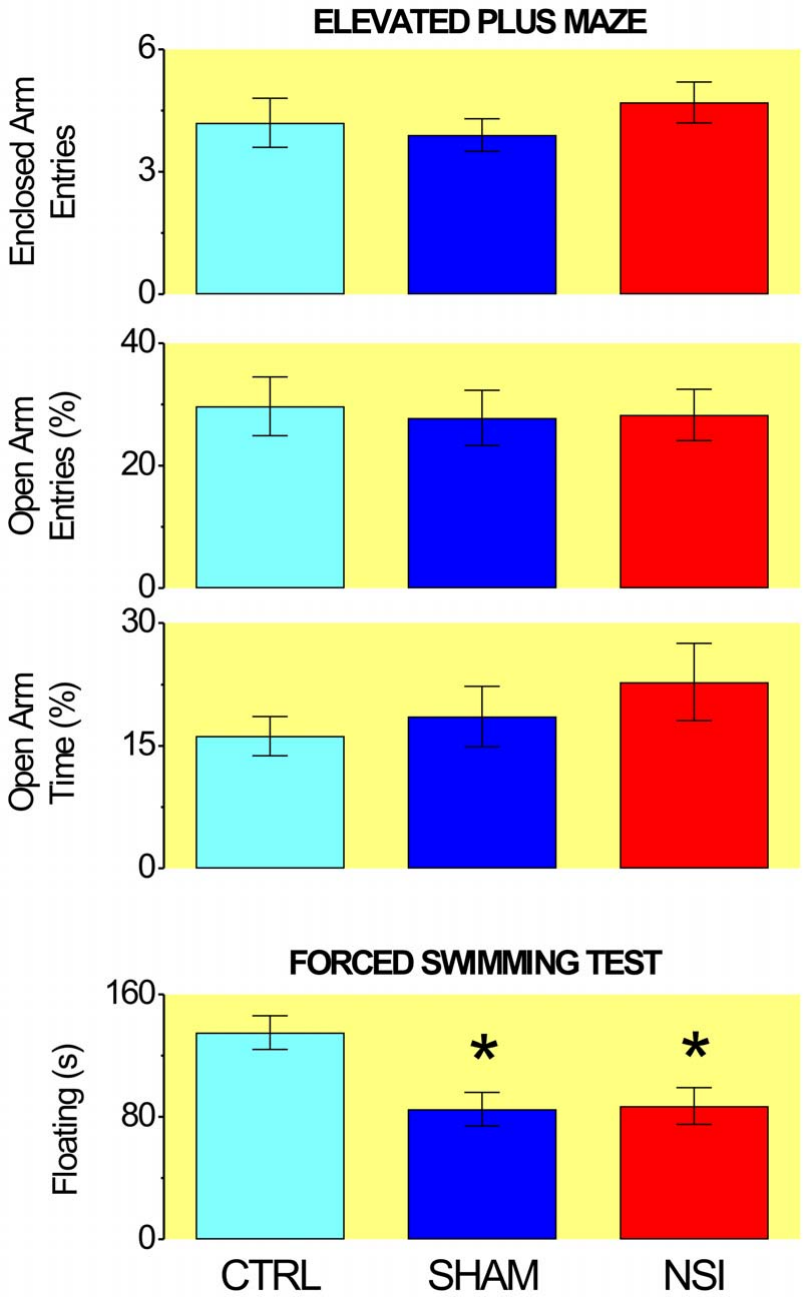
Effects of neonatal social isolation on the performance of adult rats in the elevated plus-maze (*n* = 19–27) and forced swimming test (*n* = 18–24). Columns represent mean±SEM. CTRL – controls, SHAM – sham-isolated rats, NSI – neonatally-isolated rats. EAE – number of entries in enclosed arms (mean±SEM), OAE% - percent of entries in open arms, OAT% - percent of time in open arms. * P<0.005, statistically different from CTRL.

Although the groups did not differ respecting their performance in the EPM (Fig. 4), they performed differently in the FST (F_2,58_ = 6.43, P<0.005). Differences in FST were due to the longer periods of immobility of CTRL group relative to both the NSI (t = 3.05, P<0.005) and SHAM (t = 3.16, P<0.005) groups (Fig. 4).

### NSI effects on mothering behavior

Although not quantified, it was noticed that the identification marks of isolated pups faded much faster than those of the non-separated siblings. This observation suggests more intense stroking and licking behaviors towards the reunited pup.

## Discussion

As adults, NSI rats showed marked facilitations of DPAG-evoked freezing and flight behaviors relative to both SHAM and CTRL groups. Similarly, DPAG-evoked jumping behavior was facilitated in NSI rats as compared to the former group. By contrast, groups did not differ with respect to both the defecation and micturition. The latter observations are in agreement with previous studies suggesting that DPAG-evoked defensive behaviors (freezing and flight) and pelvic viscera responses (micturition and defecation) are processed by functionally distinct systems within the PAG [8], [9], [20], [22], [45]. As it regards, it is pertinent that urges of defecation and micturition are neither experienced by patients during panic attacks [1], [2], nor recognized as symptoms typical of clinical panic [46], [47].

Group comparisons were endorsed by the remarkable similarity of brain stimulated sites. Indeed, apart from one electrode in DMPAG and three electrodes in DLSC, group stimulation sites were evenly distributed within the LPAG and DLPAG. The similarity of group stimulations is further supported by previous studies showing that current-varying sine-wave stimuli are unable to discriminate between DPAG and DLSC [20], [48]. Moreover, whereas the split-litter design makes it unlikely the influence of differences in genetic background, the remarkable similarity of SHAM and CTRL groups discards the significant effect of dam's separation stress. Consequently, group differences were most likely due to social isolation early in life.

Although not quantified, a striking observation of the present study was that the identification marks of isolated pups faded much faster than those of non-separated siblings. Because mother's licking and grooming behaviors have marked effects on pup's development [49], [50], [51], it remains unclear whether the facilitation of panic-like behaviors was due to maternal separation properly, to dam's excessive licking of reunited pup [52], or to any combination of these factors. Indeed, ultrasonic vocalizations and neuroendocrine responses of mother-deprived pups are markedly affected by dam's care giving behaviors, including feeding and anogenital stroking [53], [54], [55], and tactile and olfactory cues from both dam [56], [57], [58], [59] and siblings [60].

Present data are in agreement with previous studies showing that neonatally-isolated adult rats present sex-dependent facilitations of panic-like respiratory responses to both hypoxia (males) and hypercapnia (females) [31], [32], [33], [34], [35]. Accordingly, Kinkead and collaborators [31] suggested that NSI facilitates the central processing of chemoreceptor afferent inputs of adult rats. Yet, here we showed that NSI may also sensitize DPAG regions presumptively involved in behavioral alarm systems to both the predation (DLPAG) and suffocation (LPAG) [22]. Otherwise, NSI facilitations of behavioral and respiratory panic-like responses could be due to enduring plastic changes of PAG descending projections to cuneiform nucleus (midbrain locomotor region) and parabrachial region (pontine pneumotaxic center) respectively [21], [61], [62], [63]. Lastly, NSI panic-enhancing effects could be the outcome of early-life programming of hypothalamus-pituitary-adrenal (HPA) axis [35], [49], [50], [64]. As a matter of fact, the HPA axis is known to be hyperactive either in PD patients [65], [66] or adult rats which were subjected to 24-h mother deprivations [67], [68] or 3-h daily maternal isolations [32], [69] in the first two weeks of age.

Most importantly, however, NSI produced the selective facilitation of DPAG-evoked panic-like reactions. Indeed, whereas the anxiety and depression scores of NSI rats were virtually identical to those of SHAM rat group, depression scores of neonatally-handled groups (NSI and SHAM) were significantly reduced relative to the non-handled group (CTRL). Consequently, the augmented resilience of former groups should be credited to pup handling throughout the lactation period. Be this as it may, it remains to be elucidated whether the present results still hold in pups which isolation was restricted to the first two weeks of age comprising the “stress hyporesponsive period” [54]. In any event, our results are in line with prior studies that failed in finding any increase in basal anxiety of neonatally-isolated mice and rats which were exposed as adults to EPM, elevated T-maze (ETM), open-field (OF) and dark/light box [70], [71], [72]. Actually, there are reports of reduced levels of anxiety of neonatally-isolated adult rats exposed to EPM, ETM and OF [73], [74], [75]. Moreover, studies carried out with FST, OF and sucrose-preference anhedonia test (SPAT) did not find any sign of depression in neonatally-isolated adult rats [75], [76]. Conversely, however, a number of studies suggests that NSI rats are more anxious than controls in the EPM [75], [77], [78], [79], [80], [81]. Although the conflicting literature may be due to the many procedures of maternal separation [82], Huot and collaborators [77] reported that NSI-induced increases in scores of both anxiety (EPM) and depression (SPAT) were prevented by chronic treatment with paroxetine (7 mg/day/21 days). Similarly, unpublished results from our laboratory (C.S. Bernabé) showed that NSI-induced anhedonia was prevented by chronic administration of a clinically effective dose of fluoxetine (1 mg/kg/day/21 days). Therefore, although the evidence is mixed, the latter studies support the development of mild depressive symptoms in NSI rats in spite of the FST negative results herein reported.

Present data are reminiscent of Rachel Klein's [26] 15-year double-blind interview-based follow-up study of children displaying manifest symptoms of CSA (school-refusal). Indeed, Klein [26] found that the only significant difference in separation-anxious subjects was the increase in the rate of panic attacks in early adulthood. Yet, probands also showed a significant increase in hospitalizations due to depressive episodes and a trend (P<0.10) to an increased rate of major depression. It should be noted, however, that McGrath and collaborators [83] did not find any change in the sensitiveness to sodium lactate in non-treated depressed outpatients without PD. In the same vein, DPAG-evoked defensive behaviors were attenuated in presumptively depressed rats exposed to uncontrollable stress [84]. Accordingly, the weight of the evidence suggests that CSA predisposes subjects predominantly to PD.

Despite all the evidence to the contrary, recent studies raised serious doubts about the influence of early-life adversities on the late development of PD. Indeed, Battaglia and colleagues [85], [86], [87] showed that whereas the genetic factors are responsible for the larger proportion (64–89%) of the covariation of PD and CO_2_ hypersensitivity, environmental factors, either shared (childhood) or unique (adulthood), explained a negligible proportion of these traits if any. Similarly, Roberson-Nay and colleagues [29] showed that whereas a common genetic diathesis accounted for 39% and 35% of the respective variations of CSA and PD, shared events of childhood accounted for 1.2% of adult-onset panic attacks only. By contrast, the latter authors found that non-shared events of early adulthood were responsible for 64% of panic attacks, confirming the acknowledged importance of stressful events as triggers of PD [88], [89], [90], [91]. Because the former studies employed the same analytical procedure (Choleski's decomposition of covariance matrix) [29], [85], [86], [87], the striking difference in the contribution of adult environment may be due to the more stringent sampling criterion of Battaglia's group studies (i.e., CO_2_ hypersensitivity). In turn, the negligible contribution of childhood environment in the latter studies could be due to Choleski's procedure inappropriate assessment of gene-by-environment interactions. Indeed, studies carried out with other methods showed that CO_2_ sensitivity increases linearly with the number and severity of adverse life events [92], [93]. Moreover, Spatola and collaborators [93] found that only events which took place before 18 years of age correlated with CO_2_ hypersensitivity. These data suggest that the panic-enhancing effects of early-life adversities are solely expressed upon stressful conditions of adulthood (e.g., hypercapnia, hypoxia, trauma, loss, etc). Accordingly, NSI facilitation of DPAG-evoked panic-like responses of present study could be due to the interaction of stressful isolation periods of infancy (PN2-PN21) and adulthood (PN60-PN65 post-surgery recovery). As a matter of fact, Fournier and colleagues [94] showed that modest changes in housing of juvenile rats (pairs versus triads) may either attenuate or suppress the enhancement of respiratory responses to hypoxia in neonatally-isolated adult rats.

Concluding, whatever the mechanism involved, present data support the DPAG involvement not only in panic attacks but also in the predisposing risk of separation-anxious children to the late development of PD.
